# Case Report: Heterozygous Germline Variant in *EIF6* Additional to Biallelic *SBDS* Pathogenic Variants in a Patient With Ribosomopathy Shwachman–Diamond Syndrome

**DOI:** 10.3389/fgene.2022.896749

**Published:** 2022-08-12

**Authors:** Ibrahim Taha, Selena Foroni, Roberto Valli, Annalisa Frattini, Pamela Roccia, Giovanni Porta, Marco Zecca, Elena Bergami, Marco Cipolli, Francesco Pasquali, Cesare Danesino, Claudia Scotti, Antonella Minelli

**Affiliations:** ^1^ Department of Molecular Medicine, University of Pavia, Pavia, Italy; ^2^ Department of Medicine and Surgery, University of Insubria, Varese, Italy; ^3^ Istituto di Ricerca Genetica e Biomedica, CNR, Milano, Italy; ^4^ Pediatric Hematology/Oncology, Fondazione IRCCS Policlinico S, Matteo, Pavia, Italy; ^5^ Centro Fibrosi Cistica, Azienda Ospedaliera Universitaria Integrata Verona, Verona, Italy

**Keywords:** Shwachman–Diamond syndrome, *EIF6*, *SBDS*, whole-exome sequencing, case report

## Abstract

**Background:** Shwachman–Diamond syndrome (SDS) is a rare autosomal recessive ribosomopathy mainly characterized by exocrine pancreatic insufficiency, skeletal alterations, neutropenia, and a relevant risk of hematological transformation. At least 90% of SDS patients have pathogenic variants in *SBDS,* the first gene associated with the disease with very low allelic heterogeneity; three variants, derived from events of genetic conversion between *SBDS* and its pseudogene, *SBDSP1*, provided the alleles observed in about 62% of SDS patients.

**Methods:** We performed a reanalysis of the available WES files of a group of SDS patients with biallelic *SBDS* pathogenic variants, studying the results by next bioinformatic and protein structural analysis. Parallelly, careful clinical attention was given to the patient focused in this study.

**Results:** We found and confirmed in one SDS patient a germline heterozygous missense variant (c.100T>C; p.Phe34Leu) in the EIF6 gene. This variant, inherited from his mother, has a very low frequency, and it is predicted as pathogenic, according to several *in silico* prediction tools. The protein structural analysis also envisages the variant could reduce the binding to the nascent 60S ribosomal.

**Conclusion:** This study focused on the hypothesis that the *EIF6* germline variant mimics the effect of somatic deletions of chromosome 20, always including the locus of this gene, and similarly may rescue the ribosomal stress and ribosomal dysfunction due to *SBDS* mutations. It is likely that this rescue may contribute to the stable and not severe hematological status of the proband, but a definite answer on the role of this *EIF6* variant can be obtained only by adding a functional layer of evidence. In the future, these results are likely to be useful for selected cases in personalized medicine and therapy.

## Introduction

Shwachman–Diamond syndrome (SDS) is a rare, autosomal recessive disease (OMIM #260400) included among inherited bone marrow failure syndrome (IBMFS). It is primarily characterized by neutropenia, exocrine pancreatic insufficiency, skeletal alterations, and an increased risk to develop hematological malignancies, including myelodysplastic syndrome (MDS) and acute myeloid leukemia (AML) (Dror et al., 2011; Nelson and Myers, 2018). The disease-causing gene is *SBDS,* localized at 7q11.21 (Boocock et al., 2003), and a large majority of patients carry biallelic pathogenic variants in *SBDS*, the detection of which confirms the clinical diagnosis. Typically, mutation analysis of exon 2 identifies, in at least 90% of SDS patients, one of three common mutations, (c.183_184delTAinsCT, c.258+2T>C, and c.258+2T>C in addition to c.183_184delTAinsCT in the same allele). Two of these common mutations are observed concomitantly in approximately 62% of SDS patients (Nelson and Myers, 2018).

The approach of whole-exome sequencing (WES) has disclosed genetic heterogeneity for SDS. Indeed, biallelic mutations in DNAJ homolog subfamily C member 21 (*DNAJC21*) (Tummala et al., 2016; Dhanraj et al., 2017), in GTPase Elongation Factor-like (*EFL1*) (Stepensky et al., 2017; Tan et al., 2018; Tan et al., 2019; Lee et al., 2021), or heterozygous mutations in the 54-kDa signal recognition particle (*SRP54*) (Carapito et al., 2017) have been reported in association with SDS or SDS-like phenotype in some patients.

When *SRP54* is involved in the co-translation protein-targeting pathway*,* the other genes code for proteins involved in the ribosome biogenesis pathway as SBDS: 1) DNAJC21 is involved in the pre-rRNA processing, and it is implicated in the late 60S maturation in the cytoplasm; 2) EFL1 interacts with SBDS to evict the anti-association factor eIF6 from the ribosomal subunit 60S in the final step of ribosome maturation (Weis et al., 2015). The removal of eIF6 from the 60S subunit allows the 80S ribosome assembly (Ceci et al., 2003).

In SDS, because of the loss of SBDS activity, eIF6 remains bound to the 60S subunit and prevents its joining to the 40S subunit, resulting in decreased ribosomal subunit joining and reduced translation efficiency. The impairment of ribosome biogenesis links SDS to the group of bone marrow failure disorders, called ribosomopathies, associated with mutations in genes related to ribosomal biogenesis and function. Ribosomopathies are characterized by ribosome dysfunction producing marrow failure and cancer predisposition (De Keersmaecker et al., 2015).

Focusing on the SDS predisposition to hematological malignancies, a growing interest in eIF6 and its role in ribosome maturation prompted two different types of studies. The first concerns the interstitial deletion of the long arm of chromosome 20, del(20)(q), identified by periodic bone marrow monitoring of SDS patients; this clonal abnormality always includes the loss of the *EIF6* locus (Pressato et al., 2015). Clinical follow-up of SDS patients showed that del(20)(q), without additional clonal chromosomal abnormalities, correlates with a lower risk of developing MDS and/or AML, suggesting that the loss of one copy of *EIF6* gene restores ribosome homeostasis in the context of reduced SBDS activity (Valli et al., 2013; Khan et al., 2020; Khan et al., 2021). The del(20)(q) has also been associated with an increased number of burst-forming unit-erythroid (BFU-E) colonies in cases of paternal deletion (Nacci et al., 2017).

The second study monitored the mutational status on multiple bone marrow samples of patients with SDS. By using NGS sequencing, recent research demonstrated the frequent development of hematopoietic clones carrying a heterozygous mutation in *EIF6* and *TP53* genes. Functional studies observed the inactivation of eIF6 and the corresponding improvement of the less effective SDS translation, supporting the hypothesis of improved clone fitness (Kennedy et al., 2021; Tan et al., 2021).

All these studies unveiled that SDS represents another genetic disease in which a somatic rescue mechanism occurs, either through a del(20)(q), always encompassing the *EIF6* locus, or through *EIF6* heterozygous variants.

As a part of a larger study of whole-exome sequencing (WES) on SDS patients carrying biallelic causative *SBDS* pathogenic variants, aimed to understand if other germline variants could explain the large clinical variability among the patients (Morini et al., 2019), we identified a single case carrying a heterozygous germline variant (c.100T>C, p.Phe34Leu) in *EIF*. We postulate, with strong support from structural data, that this variant impairs protein activity, thus reducing the ribosomal dysfunction, similarly to deletions of chromosome 20, and, likely, resulting in a milder and stable hematological picture in the reported patient.

## Materials and Methods

### Case Report

Our patient, a 23-year-old male (born in 1998), identified as UPN 2 (unique patient number) in the previous work by our group (Valli et al., 2019), is the second child of healthy non-consanguineous Italian parents. UPN 2 was diagnosed as affected by SDS in 2001 based on the presence of neutropenia and pancreatic insufficiency.

Since diagnosis, an annual follow-up has been performed at the pediatric onco-hematology unit. Over the last 10 years, the patient has maintained a stable hematological picture.

At the latest clinical evaluation (2021), his height was 155.0 cm, his weight 50.0 kg, and his chest circumference 77.5 cm, all at 25–30 centiles of height and weight distribution of SDS male/female patients (personal communication by M. Cipolli and G. Tridello). At the same time, no pathological changes were observed in both lower limbs, and normal bone density was reported. Similarly, no structural changes in the thoracic cage were present. His school performance was low average. At the same clinical evaluation of 2021, hemoglobin and RBC counts were normal (14.9, normal value, nv: 13.2–17.3 g/dl and 4.35 × 10^6 per µl, nv 4.30–5.70 × 10^6 per µl, respectively). The neutrophil count was low (0.9 × 10^3, nv: 2.0–8.0 × 10^3 per µl, and ranging between 0.5 and 0.7 × 10^3 per µl in previous tests), lymphocyte count was normal (1.8 × 10^3µL, nv: 1.5–4.0 × 10^3 per µl), and platelets were slightly reduced (120 × 10^3 per µl, nv: 150–400 × 10^3 per µl, ranging between 100 and 130 × 10^3 per µl in previous tests). Bone marrow biopsy confirmed, as the previous controls, a stable cellularity (20%), and immunostaining with anti-p53 antibody was consistently negative. No evidence of any change suggesting myelodysplasia was reported.

Routine biochemical tests were within normal limits; serum amylase and pancreatic isoamylase were low, 14.0 mU/ml and 5.0 mU/ml, respectively (nv: 25.0–125.0 mU/ml and 8.0–53.0 mU/ml, respectively). Serum lipase was within normal limits: 36.0 mU/ml (nv: 8.0–58.0 mU/ml). Creon tablets as a treatment for the exocrine pancreatic insufficiency were prescribed. S-Parathyroid hormone (PTH) levels were slightly elevated during the last two checks, corresponding to 112.3 and 84.5 pg/ml values (nv: 12,0–72,0 pg/ml). Vitamin D, calcium, and potassium were at the normal levels. The patient had received his third dose of COVID-19 vaccine without any major complication.

Similarly, a recent survey carried out by an American SDS Registry, confirmed no worrying clinical effect in three SDS patients who have been vaccinated against COVID-19 (Galletta et al., 2022).

### Cytogenetics

Repeated conventional and molecular cytogenetic analyses were performed on bone marrow yearly since 2002, almost regularly, up to 2021. The methods used included routine cytogenetics, Fluorescent *In Situ* Hybridization (FISH) on metaphases and nuclei with informative probes and libraries, and array-based Comparative Genomic Hybridization (a-CGH), according to the methods reported in Pressato et al. (2015).

### Molecular and Bioinformatics Investigations

In 2015, we performed WES on 16 DNA samples obtained from blood samples of SDS patients after their acceptance to the project. Samples eligible for WES held as key features a concentration of 50 ng/ul, a ratio 260/280λ and 260/230λ, respectively, at least equal to 1.8 and 1.8–2.1, and a good integrity evaluated by agarose gel electrophoresis. Sequencing libraries of the samples were prepared using the exome enrichment kit *Agilent SureSelectXT Human All Exon V5+UTRs*. WES was performed on the HiSeq 1,000 (Illumina, 2 bp × 100 bp). The processing of sequencing data was based on the ISAAC pipeline (Raczy et al., 2013); for data alignment and for variants calling, we used, respectively, the ISAAC aligner and ISAAC variant caller. From the samples alignment we obtained a mean coverage of 65% and a coverage >20X corresponding to 91% of the complete exome sequences. For the annotation and the analysis of the variants in the variant call format (VCF) files, generated by WES for each sample, the expert variant interpreter (eVai) platform (https://www.engenome.com/evai/) (Nicora et al., 2022) was used.

We confirmed the relevant variants by Sanger sequencing. The primers used for the amplification and sequencing were designed using Primer3web (https://primer3.ut.ee/). PCRs were performed in 25 μL of reaction volume containing 50 ng of genomic DNA, 0.5 μm primers, 2 μl dNTP Mixture (2.5 mm each), 2.5 μl 5X PrimeSTAR GXL Buffer, and 1.25U PrimeSTAR GXL DNA Polymerase (TAKARA BIO INC.). PCR products were checked by 2% agarose gel electrophoresis and underwent clean-up prior to sequencing using the Applied 3500 DX Genetic Analyzer sequencer with BrilliantDye Terminator Cycle sequencing kit (NimaGen). The sequencing data were analyzed by Finch TV software.

### Structural Analysis

For structural visualization and evaluation of the effect of the Phe34Leu mutation, the model of eIF6 was extracted from PDB code 6LSS (pre-60 ribosome). This model had originally been generated by SWISS-MODEL (Waterhouse et al., 2018) and docked into the electron density of human pre-60S ribosomal particles by rigid-body fitting, followed by extensive manual rebuilding in COOT with a final local resolution comprised between 3.1 and 4.8 Å (Liang et al., 2020).

## Results

### Cytogenetic Findings

Bone marrow karyotype analysis was normal up to 2007; then, from 2008 to 2010 a small clone was detected carrying an isochromosome for the long arm of chromosome 7, i(7)(q10), which is one of the most frequent clonal chromosome anomalies in the bone marrow of SDS patients: the abnormal cells ranged from 1.7 to 7.6%, according to different methods (conventional cytogenetics or FISH with different probes) or different times of analysis. On DNA from a sample with 2.9% of cells with the i(7)(q10), a-CGH was performed; it did not reveal the chromosome 7 abnormality due to the small size of the clone, nor any other abnormality. The i(7)(q10) clone then disappeared, and was not found in any subsequent analysis from 2011 to 2020; no structural abnormality of chromosome 20 was ever identified.

### Molecular and Bioinformatics Analysis

The clinical diagnosis of SDS was confirmed by the presence of one of the common mutations [c.258+2T>C, p.Cys84fs3, paternal] and a second variant [c.356G>A, p.Cys119Tyr, maternal] in *SBDS*. The latter was included by Finch et al. (2011) among disease-associated missense variants since, on the basis of functional studies, it was evaluated as a mutation affecting SBDS protein stability.

**FIGURE 1:**
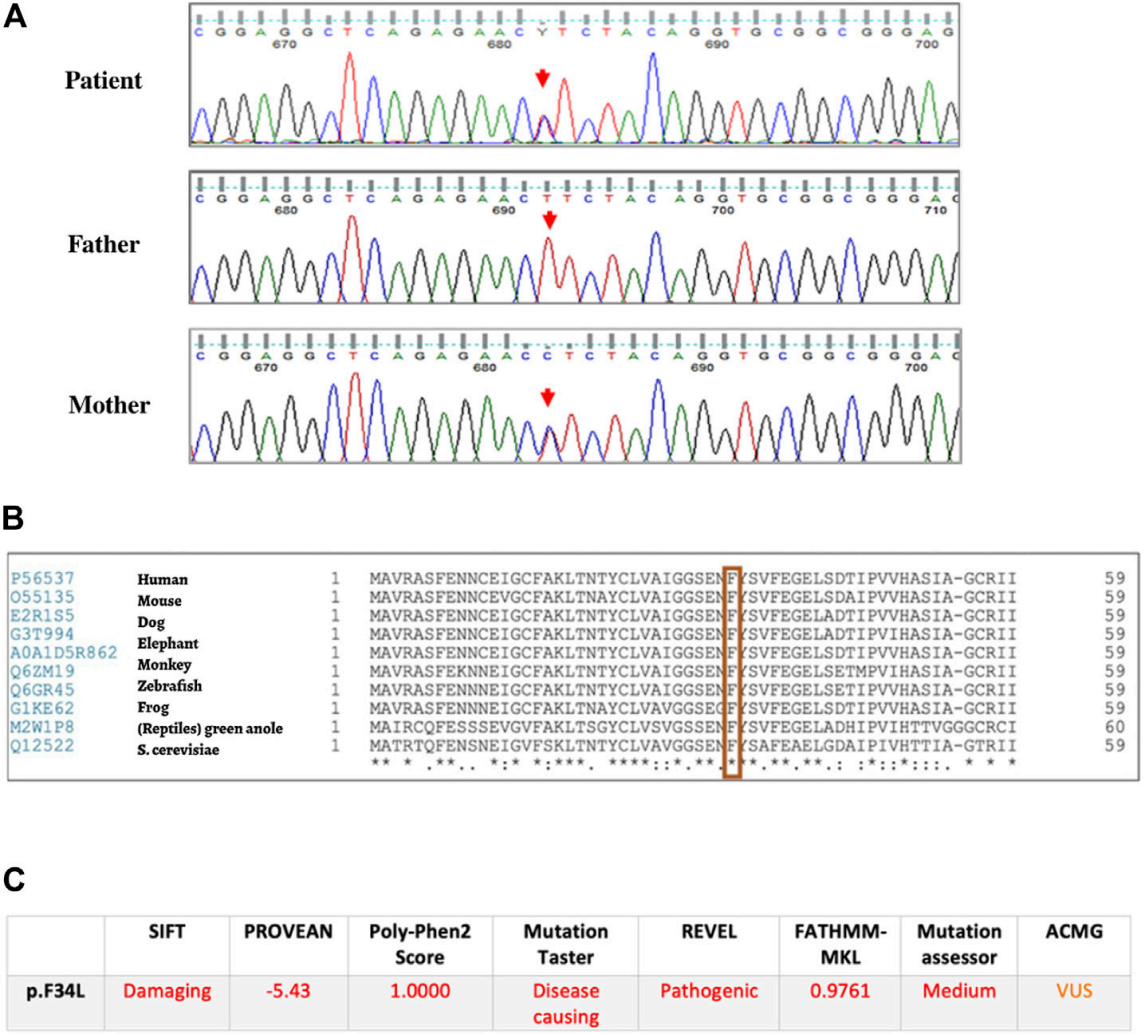
Sequencing confirmation of the *EIF6* variant. **(A)** Part of electropherogram from the proband and their parents, showing the substitution T>C in the heterozygous status at the position c.100 maternal inherited; **(B)** protein sequence alignment using UniProt shows absolute conservation of F34 throughout different species (human, mouse, dog, elephant, monkey, zebrafish, frog, reptiles, and *S. cerevisiae*); **(C)** *in silico* analysis of the p.Phe43Leu variant using different prediction tools.

When reviewing our WES file of UPN2 using the eVai platform, we first filtered the results searching for variants in other genes related to SDS or SDS-like phenotypes, *DNAJC21*, *EFL1*, and *SRP54*, no relevant variants were found. Then, checking for variants in *EIF6* gene, we found a heterozygous single nucleotide substitution causing a missense change (c.100T>C; p.Phe34Leu). Sanger sequencing confirmed the variant for the patient and assessed its maternal origin (Figure 1A). According to the Genome Aggregation Database (gnomAD), the variant is a rare one as its frequency is 0.0002002.

These results were obtained in DNA from a blood sample, but we checked the variant in DNAs also from eight bone marrow specimens obtained at regular follow-ups (the latest one in 2021): all of these showed the stable presence of the heterozygous variant, with a ratio 1:1 to normal allele.

Protein sequence alignment using UniProt (www.uniprot.org) showed that the amino acid Phenylalanine in position 34 is highly conserved among different species such as human, mouse, dog, elephant, monkey, zebrafish, frog, reptiles, and *S. cerevisiae* (Figure 1B). The variant functional predictions are entered in Figure 1C.

We then filtered the WES results for the presence of variants in genes related to the development of MDS or leukemia, as reported by several authors (Lindsley, 2017; Steensma, 2017; Armstrong et al., 2018; Bluteau et al., 2018; Obrochta and Godley, 2018; Kennedy et al., 2021). The variants with a frequency ≤0.01 were validated by Sanger sequencing in the patient and his parents. These variants are entered in Table1 with their genome position, amino acid change, zygosity, allele frequency, and pathogenicity prediction, according to various specific tools and parental origin.

**TABLE 1:** Variants found in UPN 2 with a frequency ≤0.01 in genes related to MDS and AML.

Chr: Position	Gene	cDNA (a.a)	Effect	Zyg	MAF	*In silico* prediction tools							Parental Origin
						SIFT	Mutation assessor	FATHMM	MutationTaster	M-CAP	REVEL	ACMG		
20:31,024,146	ASXL1	c.3631G>A (p.Asp1211Asn)	Missense	Het	0.0	Damaging	Medium	Tolerated	Polymorphism	Tolerated	Benign	VUS	Father
9:5,022,031	JAK2	c.44C>T (p.Ser15Phe)	Missense	Het	0.0000159	Damaging	Low	Tolerated	Polymorphism	Possibly pathogenic	Benign	VUS	Father
20:57,474,003	GNAS	c.2149G > T (*p*.Glu717Ter)	LoF	Het	0.0	NA	NA	Damaging	Disease causing	Possibly pathogenic	NA	Pathogenic	Father

MDS, myelodysplastic syndromes; AML, acute myeloid leukemia; Chr, chromosomal; A.A, amino acid change; LoF, loss of function; Zyg, zygosity; Het, heterozygous; MAF, minor allele frequency; VUS, variant of uncertain significance; SIFT, sorting intolerant from tolerant (https://sift.bii.a-star.edu.sg); FATHMM, functional analysis through hidden Markov models (http://fathmm.biocompute.org.uk); M-CAP, Mendelian clinically applicable pathogenicity (http://bejerano.stanford.edu/mcap); REVEL, rare exome variant ensemble learner (https://sites.google.com/site/revelgenomics); ACMG, American College of Medical Genetics

### Structural Analysis

**FIGURE 2:**
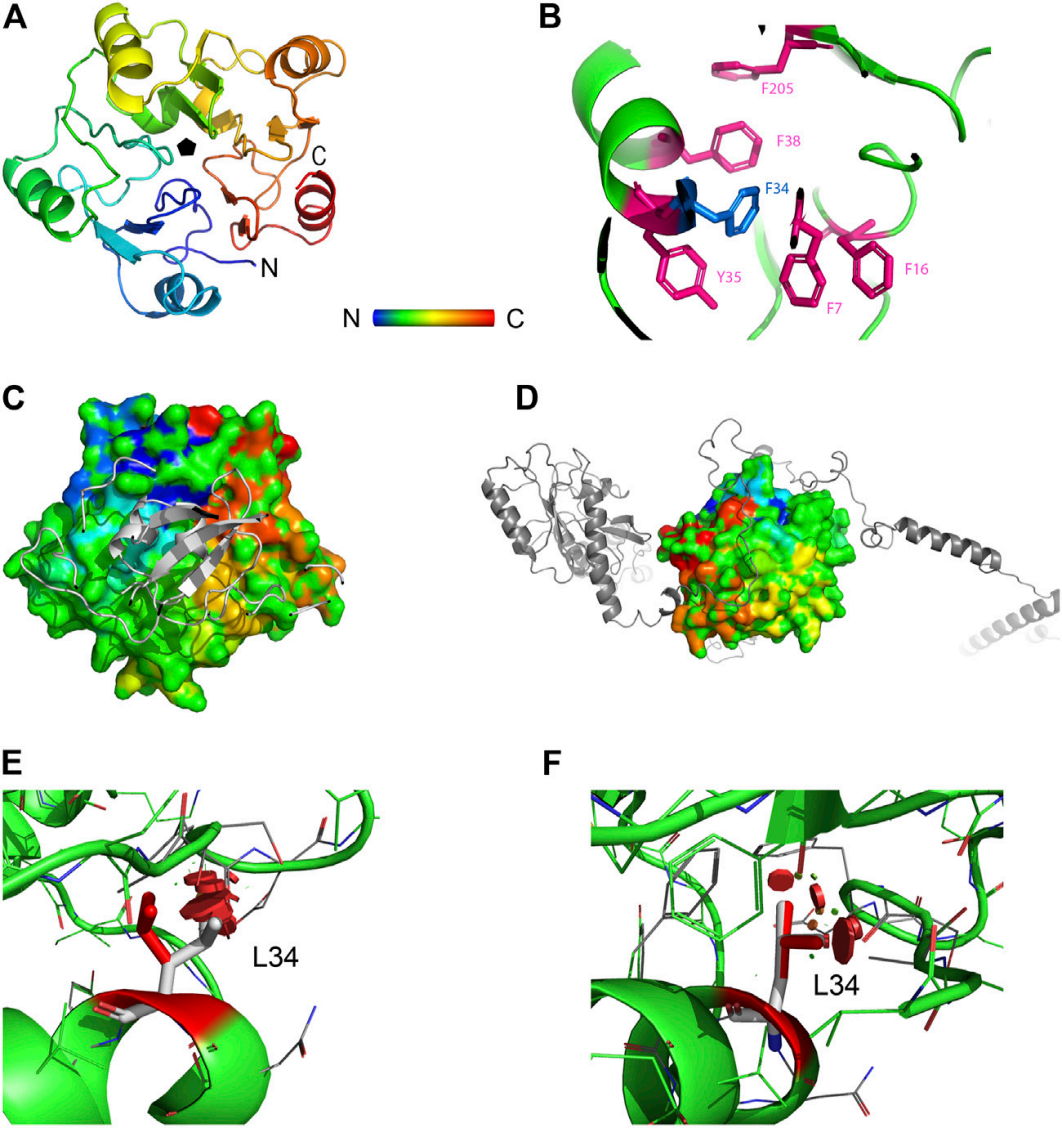
Structural analysis of eIF6. **(A)** Rainbow-colored (blue: N-terminus, red: C-terminus) ribbon representation of eIF6, consisting of five quasi-identical alpha/beta subdomains arrayed about a five-fold axis of pseudosymmetry. **(B)** Residue F34 (blue sticks) is the core part of a chain of hydrophobic interactions mediated by a cluster of five aromatic residues (F7, F16, F34, F38, and Y35) belonging to the N-terminal domain and one, F205, belonging to the C-terminal domain (pink sticks). **(C)** Surface representation of eIF6 interacting with ribosomal protein L23 (gray ribbon). **(D)** Surface representation of eIF6 interacting with ribosomal protein GTPBP4 (gray ribbon). **(E,F)** Effect of the F34L mutation: red solid disks represent clashes between the two rotamers of L34 and neighboring residues.

The protein eIF6 consists of five *quasi*-identical alpha/beta subdomains arrayed about a five-fold axis of pseudosymmetry (Figure 2A). Residue F34 (blue sticks) is the core part of a chain of hydrophobic interactions mediated by a cluster of five aromatic residues (F7, F16, F34, Y35, and F38) (pink sticks) belonging to the N-terminal domain and one, F205, belonging to the C-terminal domain (Figure 2B). This residue arrangement, involving helix 2A and β-strand 2E, where the group of four residues, F34, Y35, F38, and F205, are located, respectively (nomenclature according to (Groft et al., 2000), contributes to stabilize the “velcro” strategy adopted in the pentein fold of eIF6 (Fülöp and Jones, 1999), where β-strand 2E at the C-terminus hydrogen bonds with a β-strand from the N-terminal subdomain. The flat protein surfaces, thus, formed are necessary for interaction with ribosomal protein L23, a component of the 60S subunit, on one side, and with GTPBP4 (nucleolar GTP-binding protein 1), also involved in the 60S biogenesis, on the other (Figures 2C,D). The F34L variant lacks the central phenylalanine of the cluster, interrupts this regular pattern and introduces clashes, represented by solid red disks in Figures 2E,F, where both leucine rotamers are considered. Despite the similar hydrophobic nature of the two residues, the reduction of the side chain size and the interruption of the aromatic stack explain why analysis by I-mutant predicts a Gibbs free energy difference (ΔΔG) between the mutant and the wild-type of −0.87 kcal/mol. This result is evidence of a large decrease in protein stability. We can, therefore, envisage that the F34L variant could impair eIF6 folding, with a reduction in binding to the nascent 60S ribosomal subunit through protein L23 and a possible consequent reduction in binding of protein GTPBP4, which is also involved in ribosomal biogenesis.

## Discussion

Three main features characterize SDS: bone marrow failure, pancreatic exocrine dysfunction, and skeletal abnormalities (Dror et al., 2011). In addition, a significant percentage of SDS patients may develop serious hematological complications, requiring, in some cases, hematopoietic stem cell transplantation, a procedure associated with a requirement for specific protocols for this disease (Donadieu et al., 2012). A challenge for the surveillance of the disease is the early identification of risk factors relevant to the evolution of the hematological picture (Furutani et al., 2022). An extensive study on a French cohort of 102 patients postulated that the combination of a young age at diagnosis with low values of specific hematologic parameters, at the time of diagnosis and later constitutes a negative prognostic factor. This combination was a risk factor stronger than any of the other nine single variables taken into consideration alone, as transient cytopenia, low value of neutrophils, hemoglobin, platelets, or *SBDS* genotype. The latter was confirmed not to be linked to significant differences in hematologic parameters and other nonhematologic features, both in patients harboring two common mutations or in patients with one common and one rare mutation (Donadieu et al., 2012).

Although the risk for developing MDS and AML is not related to the type of *SBDS* mutations, it can be favorably modified by other genetic factors as the presence of specific chromosomal abnormalities, in particular, the i(7)(q10) and del(20)(q) if they are the only abnormality present (Minelli et al., 2009; Khan et al., 2021).

When del(20)(q) is considered, cytogenetic studies, made over a long period of time on the SDS Italian cohort, confirmed the benign prognostic significance of this abnormality. Indeed, the patients carrying del(20)(q) consistently showed a mild hypoplastic bone marrow picture, no or mild neutropenia, anemia, and thrombocytopenia (Pressato et al., 2012; Valli et al., 2019).

In all cases, del(20)(q) includes the loss of the *EIF6* locus and, consequently, in the bone marrow clones with the chromosomal abnormality, only one allele expressing *EIF6* is present in the bone marrow clones with the chromosomal abnormality. This condition has been studied *in vivo* on mice demonstrating that knockout heterozygous animals presented reduced eIF6 levels as expected, while preserving sufficient nucleolar eIF6 and regular ribosome biogenesis. In the liver cells, a larger amount of 80S in polysomal profiles suggested a lower efficiency in initiation of translation (Gandin et al., 2008).

Common events in SDS patients are the development of many somatic clones in the bone marrow with variants in either *EIF6* or *TP53* genes, producing different effects: i) variants in *EIF6* have a compensatory role on the impact of SBDS deficiency, in turn reducing the risk of developing MDS and AML by improving the ribosome maturation, enhancing translation, and reducing p53 upregulation (Kennedy et al., 2021), ii) variants in *TP53* act in a different pathway and decrease tumor suppressor checkpoint activation. Clones acquiring only variants in both *TP53* alleles are associated with the development of hematological malignancies (Kennedy et al., 2021).

Furthermore, higher p53 protein levels, linked to inhibition of osteogenesis, have been found in SDS patient’s osteoblasts, in correlation with the skeletal anomalies of patients (Frattini et al., 2021).

Relevantly, the *EIF6* clonal variants in SDS patients were not observed in the presence of leukemic evolution or serious bone marrow dysfunction, confirming a positive impact on the hematological alterations determined by the *SBDS* genotype (Kennedy et al., 2021).

The results on humans fit the yeast model proposed by Menne et al. (2007) who demonstrated that missense variants in *Tif6* can bypass the fitness defect resulting from a lack of *Sdo1* through the reduction of *Tif6* binding to the 60S ribosome subunit.

Here, we report a patient, UPN 2, in whom we found a heterozygous germline variant, c.T>C (p.Phe34Leu), in *EIF6.* This variant is rare according to gnomAD and the amino acid Phe34 is extensively conserved (Figure 1B). To the best of our knowledge, this is the first patient in whom a constitutional pathogenic *EIF6* variant is associated with biallelic *SBDS* mutations. In UPN 2, the missense variant in *EIF6* is a germline variant thus, at least in theory, its effect in ameliorating the consequences of *SBDS* mutations is expected to have started to act from zygote and to be widespread to all tissues.

Available clinical data suggest that stature (the patient is at low centiles for SDS patients), pancreatic function (the patient is still on therapy with pancreatic enzymes), and cognitive development are not strongly and favorably influenced. As far as the skeletal features are concerned, osteoblasts from UPN 2 did not differ with respect to osteoblasts from a group of 13 SDS patients studied by Frattini et al. (2021) who demonstrated lower mineralization capacity and lower expression of genes responsible for osteoblastogenesis.

Conversely, hematological data and bone marrow picture, over the last 10 years, have been stable; hemoglobin, platelets, and lymphocytes have consistently been within normal limits and the patient never needed any kind of therapy. The patient has moderate cytopenia, which does not appear to have ever required transfusions or the use of hematopoietic growth factors.

Only mild bone marrow alterations were present, and no evidence of any worsening has been reported so far, during the course of the disease. In addition, negative anti-p53 antibody reactivity has been repeatedly observed.

Similarly, Tan et al. (2021) reported an *SBDS*-mutated patient with a somatic bone marrow variant in *EIF6* (c.182G>T, p.Arg61Leu), showing stable and mild hematological features. When studying this patient, they interpreted the variant as an example of a somatic genetic rescue. The Arg61Leu variant in fact rescued the defect of Sdo1-deficient yeast cells and the larval lethality of Sbds-deficient *Drosophila* (Tan et al., 2021). We expected that the variant p.Phe34Leu, identified in our patient, could have a similar effect as Arg61Leu, predicting a reduced binding to the nascent 60S ribosome, but it needs to be proven.

Our findings, on a germinal level, might agree with the hypothesis that somatic *EIF6* mutations, observed by Kennedy and others, produce a compensatory effect for the pre-existing germline *SBDS* mutations, thus reducing the risk of developing malignant neoplasia. Among these somatic mutations, missense ones were predominantly located in regions encoding conserved secondary structure motives, exactly like the p.Phe34Leu variant described here, which is a newly described one (Kennedy et al., 2021).

In all SDS patients, the diagnosis prompts compulsory monitoring, over time, of the most relevant symptoms, including exocrine pancreatic dysfunction, especially bone marrow failure (Valli et al., 2017). In our case, this program of surveillance will produce a large amount of useful information not only to manage the clinical problems of the patient but also to compare him with the group of other SDS patients, whose exome analysis did not reveal any significant *EIF6* variants.

If the periodic clinical check will confirm a stable and positive situation, particularly concerning the hematological picture, we can confirm that we have observed a human example of somatic genetic rescue through *EIF6* mutations comparable to yeast and *Drosophila* models.

Undoubtedly, our observations, supported by genetic considerations and *in silico* simulations, may be of actual use for clinical interpretation only when experimentally validated.

The study of the effect of p.Phe34Leu variant could also be improved by using the molecular dynamics simulation approach, as previously performed for EFL1 (Delre et al., 2020).

To check the presence, in addition to the *EIF6* variant, of any other variants in genes involved in MDS or AML (Lindsley, 2017; Steensma, 2017; Armstrong et al., 2018; Bluteau et al., 2018; Obrochta and Godley, 2018; Kennedy et al., 2021), we analyzed the exome file of UPN 2 and three genes, *ASXL1*, *JAK2*, and *GNAS*, were identified as bearing a mutation which might be relevant for the risk of MDS/AML (Table1).

***ASXL1***: Several studies showed that *ASXL1* somatic mutations are among the most frequent variants in all subtypes of myeloid malignancies including MDS (11–21% cases), AML (5–11%), myeloproliferative neoplasm (MPN), and chronic myelomonocytic leukemia (CMML) (40–50%) (Boultwood et al., 2010; Thol et al., 2011; Fujino and Kitamura, 2020). *ASXL1* mutations accelerate myeloid malignancies and are always associated with poor prognosis (Gelsi-Boyer et al., 2012).

Confirmatory evidence of *ASXL1* relevance in hematological malignancies was produced by Wang et al. (2014) who showed that heterozygous genetic *ASXL1*-knockout mice (Asxl1^+/−^) developed MDS/MPN and by Abdel-Wahab et al. (2013), who reported that hematopoietic cell-specific deletions of Asxl1 induced an MDS-like disease. Germinal *de novo* nonsense or frameshift mutations in *ASXL1* cause the Bohring–Opitz syndrome (Bedoukian et al., 2018), and a few reports describe missense germline mutations, in association with familial recurrence of different hematological malignancies.

Hamadou and coworkers reported the mutation p.Arg402Gln in two Tunisian sisters affected by NHL (tonsil large B-cell lymphoma and gastric MALT lymphoma) (Hamadou et al., 2016); Seiter and coworkers reported a father and son, both carrying the p.Asn968Ser mutation, who developed MDS then evolving in AML at age 75 and 46, respectively (Seiter et al., 2018).

Zebisch and coworkers described a family in which a 60-year-old female affected with MDS was transplanted from her 58-year-old apparently healthy sister; the graft function was poor, and laboratory investigations demonstrated the presence of the same *ASXL1* mutation, p.Pro808His, in the donor sister (Zebisch et al., 2020).

An *ASXL1* mutation p.Gly704Arg is present in a family (a 2-year-old child and her 54-year-old paternal uncle, both affected with primary myelofibrosis) included in the study of Andres-Zayas and coworkers on germline predisposition for leukemia (Andrés-Zayas et al., 2021).

In our patient, the missense variant p.Asp1211Asn is interpreted as pathogenic only by SIFT (https://sift.bii.a-star.edu.sg) (Vaser et al., 2016) (Table1), and the previously reported germinal *ASXL1* mutations were reported as damaging or disease causing by SIFT and MutationTaster, respectively (https://www.mutationtaster.org) (Schwarz et al., 2014), while other tools classified them as benign.

Based on available evidence, missense germinal mutations in *ASXL1* might act as a risk factor for developing hematological malignancies; topics such as penetrance or anticipation can be discussed only when a much larger number of cases have been studied (Seiter et al., 2018). At present hematological surveillance in carriers of germinal missense mutations in *ASXL1* seems a reasonable management of the problem, even if the functional significance of the mutation is not fully defined.

***JAK2***: The *JAK2* variant, p.Ser15Phe, is interpreted as pathogenic only in one out of the seven prediction tools used (Table 1); it falls at the N-terminus outside the most functionally relevant domains reported by Benton and coworkers (Benton et al., 2019). Reports about germinal *JAK2* mutations are rare (Goldin et al., 2009; Park et al., 2020) and quoted mutations are described to be pathogenic when present in bone marrow as somatic mutations (Klampfl et al., 2013; Tefferi, 2016).

***GNAS***: The *GNAS* variant p.Glu717Ter, found in our patient and paternally inherited, is interpreted as pathogenic by all four applicable prediction tools (Table1). Also, for *GNAS* reported evidence underlines its involvement in both genetic and hematological diseases.

Activating gain-of-function mutations are known to cause Mendelian disorders as polyostotic fibrous dysplasia and McCune–Albright syndrome (Robinson et al., 2016). *GNAS* somatic mutations have been recurrently found in hematological disorders such as MDS and lymphoma (Bejar et al., 2011; Xie et al., 2014). In addition, *GNAS* germline mutations are related to different types of pseudohypoparathyroidism (PHP-Ia,-Ib,-Ic) and pseudopseudohypoparathyroidism (PPHP). PHP and PPHP are autosomal dominant conditions and are associated with a lack of expression of Gsα if the pathogenic variant inactivates maternal or paternal alleles, respectively (Haldeman-Englert et al., 1993). As the clinical data on our patient include increased PTH, a careful evaluation for endocrinological condition was suggested to the family, and it is still in progress.

## Conclusion

UPN 2 was diagnosed with SDS by targeted mutation analysis. WES studies for variants known in hematological disorders identified a variant in *EIF6* which is likely to be associated with a favorable bone marrow condition, a variant in *ASXL1* which warrants hematological surveillance, already scheduled in any SDS patient, and a variant in *GNAS*, which may explain the endocrinological abnormalities found, which are not part of the description of the syndrome.

These types of data, especially if it is supported by functional studies, clearly suggest that the use of extended genome analysis can provide useful data for better patient management.

This was demonstrated here for SDS, but it may be extended to other disorders. Certainly, a consistent portion of phenotypic variability in patients sharing the same monogenic disease is caused by additional genomic alterations. The progress in identifying these changes will help walk on the path of personalized medicine.

## Data Availability

The datasets for this article are not publicly available due to concerns regarding participant/patient anonymity. Requests to access the datasets should be directed to the corresponding author.
